# Wormholes in Host Defense: How Helminths Manipulate Host Tissues to Survive and Reproduce

**DOI:** 10.1371/journal.ppat.1004014

**Published:** 2014-04-17

**Authors:** Deborah Boyett, Michael H. Hsieh

**Affiliations:** 1 Program in Human Biology, Stanford University, Stanford, California, United States of America; 2 Department of Urology, Stanford University School of Medicine, Stanford, California, United States of America; University of Wisconsin Medical School, United States of America

Over 1,000,000,000 people and innumerable other animals are currently infected with one or more helminths [1]. These highly prevalent infections contribute to significant illness and economic losses due to impaired worker productivity and livestock health. As such, it is tremendously important to vaccine and anthelminthic drug development efforts to understand the complex interactions between host and parasite, including helminth manipulation of host tissues. The extensive coevolution of helminths and their hosts often blurs the line between what is host-mediated and what is parasite-driven. However, we have drawn explicit examples from the literature to highlight five major themes of helminth manipulation of host tissues: disruption of epithelial barriers, reinforcement of epithelial barriers, alteration of lymphoid tissue, modulation of tissue vascularity, and tuning of granulomatous responses. Herein we focus on examples of direct molecular level effects on host tissue to highlight strategies common to numerous helminths in their efforts to survive and reproduce within their hosts.

## Disruption of Epithelial Barriers

**Figure 1:**
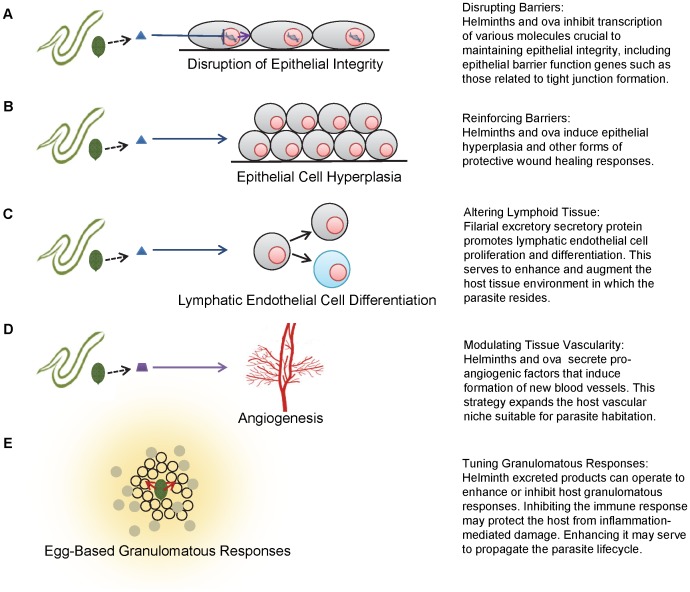
Schematics of various strategies employed by helminths to modulate host tissues. A, disruption of epithelial integrity. B, epithelial cell hyperplasia. C, lymphatic endothelial cell differentiation. D, angiogenesis. E, egg-based granulomatous responses.

A major tool in the helminth arsenal is the ability to compromise the host epithelium, which is the first line of defense against parasitic infection. For instance, schistosomes encounter numerous epithelial barriers in their life cycle. These barriers include the skin during its invasion by cercariae, the lung during schistosomular transit, and the intestine and bladder during egress of schistosome eggs in the fecal and urinary streams, respectively. Based on in vitro studies, schistosomal cercariae are thought to employ proteases such as cercarial elastase to degrade human skin elastin and collagen during the first stage of infection, allowing penetration of skin epithelium [2]–[4]. When schistosome worms mature, mate, and lay eggs, it becomes essential for the eggs to exit the host body, as the parasite life cycle will not continue without a secondary host. *Schistosoma haematobium* eggs, in particular, have developed molecular strategies for easing passage from the bladder tissue to the urinary stream (Figure 1). Mouse bladders injected with *S. haematobium* ova exhibit decreased transcription of all uroplakin genes and various tight junction related genes [5]. The structural proteins that these genes encode are essential for the maintenance of bladder epithelial integrity and the sequestration of urine from tissues extraneous to the bladder. The altered transcription of these genes may benefit helminth survival by facilitating access to the bladder lumen and the next stage of the life cycle.

In contrast to schistosomes, or blood flukes, which aggressively invade their host through the skin, many gastrointestinal helminths traverse host epithelia after being ingested. The whipworm, *Trichuris trichiura*, may alter host intestinal epithelial cells using secretory proteins. It is postulated that these proteins enable the worm to form ion conduction pores in the lipid bilayer of cecal epithelial cells, allowing the parasite to burrow its head into a nutrient-rich, intracellular microenvironment, and providing shelter from the mechanical movements of gut tissue [6]–[8].

Likewise, *Strongyloides stercoralis* perturbs mucosal integrity by disrupting the balance between cell death and proliferation in the small intestine. Infection with this helminth induces increased apoptosis and inhibits cell proliferation in the duodenum and upper jejunum, the sites of adult parasite residence [9]. A compromised intestinal barrier may facilitate the autoinfection phase of *St. stercoralis* infection, in which the rabtidiform larvae penetrate the intestinal mucosa to perpetuate infection.

Thus, diverse helminths exhibit the common strategies of compromising host epithelia in order to more successfully feed, remain in their preferred anatomic niches, and complete their life cycles.

## Reinforcement of Epithelial Barriers

In addition to breaching host epithelia to facilitate infection, helminths reinforce epithelial barriers to reduce host morbidity and thereby promote parasite survival. It is conjectured that *S. japonicum* ova have the ability to induce increased impermeability of the colonic epithelial barrier, which diminishes bacterial translocation and associated host morbidity and mortality. This reinforcement of the epithelial barrier is likely mediated by an egg-triggered increase in occludin, an integral tight junction protein [10].

Similarly, it is possible that the presence of *S. haematobium* eggs in the host bladder triggers hyperplasia of the urothelium as part of a protective resealing response to ova-induced tissue damage and concurrent inflammation [5]. Non-parasite-related in vitro studies have shown that hyperplastic urothelium exhibits greater transepithelial resistance than normal urothelium. However, cells comprising this modified barrier are less differentiated, suggesting that this is a transient phenomenon associated with urothelial repair [11]. The hyperplastic response is likely employed to rapidly reestablish and maintain transepithelial resistance, allowing time for urothelial cells to sufficiently differentiate and complete restoration of the urothelial barrier. Besides trematodes, this hyperplastic implementation has been demonstrated in a variety of helminthic infections, including the nematodes *Trichinella spiralis*, *Nippostrongylus brasiliensis*, and *Heligmosomoides polygyrus* (Figure 1) [12]. Despite numerous examples of parasite-associated epithelial hyperplasia, it remains unclear if the hyperplastic response is parasite- or host-mediated.

## Alteration of Lymphoid Tissue

The exploitation of host immune structures is a particularly striking example of the adaptive mechanisms helminths have evolved to bypass host defenses. It has been proposed that *S. mansoni* worms remodel the vasculature and structure of Peyer's patches in the gut, rendering this tissue more suitable for egg deposition and excretion [13]. Modification of the high endothelial venules supplying Peyer's patches increases the amount of eggs that can be accommodated in these lymphoid tissues. Furthermore, *S. mansoni* ova are cytotoxic to Peyer's patch fibroblasts, subsequently reducing overall stromal and lymphocyte cellularity. These methods may work in concert to facilitate egg excretion into the fecal stream and the resulting propagation of the worm life cycle.

Filarial nematodes also alter lymphatic tissue, possibly to transform these host structures into a more suitable microenvironmental niche. Excretory secretory products from *Brugia malayi* have been demonstrated to induce lymphangiogenesis, promoting lymphatic epithelial cell proliferation and differentiation (Figure 1) [14]. This effect is lymphoid tissue-specific and possibly not mediated by conventional adaptive immune processes, since severe combined immunodeficiency (SCID) mice deficient in B and T cells also exhibit lymphatic dilation in response to filarial infection [15]. This study, however, does not exclude the involvement of the innate immune response.

## Modulation of Tissue Vascularity

Helminth modulation of vasculature is also a potent mechanism by which worms control host tissues to generate favorable microenvironmental niches. *Dirofilaria immitis* infection results in canine and feline cardiopulmonary dirofilariosis due to parasite residence in the pulmonary arteries and right ventricle. As such, *D. immitis* excretory secretory antigens directly affect vascular endothelial cells, inducing vasodilation and decreasing endothelial barrier permeability to monocytes [16]. These effects have the potential to greatly benefit the parasite, as the dilation of blood vessels enables the host to accommodate additional filaria, and the reduction in monocyte mobility allows for control of perivascular inflammation, enhancing the longevity of the host and parasite.

The theme of parasite-associated vascular remodeling is further exemplified by *T. spiralis* invasion of human host musculature. In order to maintain chronic infection, it is hypothesized that *T. spiralis* larvae transform resident myocytes into nurse cells, complexes that protect the parasite from host immune responses [17], [18]. Nurse cells are innervated by a complex system of venules, hypothesized to be the result of *T. spiralis*-mediated angiogenesis (Figure 1). The induced vasculature provides the parasite with a constant supply of nutrients as well as a reliable waste disposal system. During the early stages of infection, elevated expression of vascular endothelial growth factor (VEGF) can be observed in nurse cells, despite the absence of a hypoxic event which, in the majority of situations, initiates the formation of new vessels. This altered VEGF expression may implicate a parasite-mediated effect, although the precise mechanism by which larvae directly induce angiogenesis has not been elucidated.

It is noteworthy that cancers also employ a variety of strategies to induce angiogenesis and hypervascularity. The amplified production of VEGF by neoplasms allows for a sustained and adequate supply of nutrients and oxygen that intensify tumor growth [19]. Clearly, methods of co-opting tissue vascularity are not limited to helminths, and the elucidation of the parallel mechanisms by which parasites and cancers, in essence “self”-derived parasites, accomplish these tasks should be of interest to parasitologists and tumor biologists alike.

## Tuning of Granulomatous Responses

Finally, helminths are able to modulate host granuloma development, one of the most prototypical mammalian responses to chronic infection. Schistosome ova require the formation of granulomas to translocate from the gut and bladder to their respective lumens [20], [21]. In fact, *S. mansoni* egg excretion is reduced and mortality markedly increased in mice lacking a granulomatous response [22]. To ensure that the host immune response will adopt the necessary Type 2 phenotype, schistosome worms have the ability to prime immune cells toward the Type 2 environment before egg deposition [23]. Furthermore, schistosome ova themselves may directly affect granuloma comprising cells (Figure 1): alternatively activated macrophages primed by secreted egg antigen down-regulate Type 1 immune responses, decreasing inflammation-associated mortality [24], [25], and *S. mansoni* eggs are effective inducers of Type 2 responses [26]. Thus, while the granuloma benefits the parasite life cycle, it may also protect the host from the damaging effects of immune hyperresponsiveness.

This down-regulation of the potentially harmful host immune response is repeatedly conserved across helminth-host interactions. It is conjectured that the cestode *Taenia solium* also actively down-regulates Type 1 host granulomatous responses, employing excretory secretory proteins to alter host mRNA expression of pro-inflammatory cytokines [27]. The viable cysticerci seem to have the ability to guide the immune system towards a Type 2 response, potentially decreasing inflammation and reducing damage to the parasite. Tellingly, it is only when metacestodes have begun to degenerate that disease pathology manifests [28], [29], highlighting the active, parasitic modulation of host-driven granuloma formation.

## Conclusion

Helminths evade, hijack, and modulate host immunity, often to the benefit of both the helminth and infected host. Clarifying the prolific methods that parasite worms employ to manipulate their hosts will allow us to more fully understand tissue pathogenesis, parasite transmission, and the immune system more broadly. Cancer biologists may also take interest in this line of research, as tumors employ similar mechanisms to suppress host immune function and promote cancer progression through increased vascular permeability and angiogenesis [19], [30]. Through work along these lines of inquiry, we can hasten the eradication of helminthic diseases altogether, one of the great scourges of human and animal health worldwide.

## References

[1] Hotez PJ, Brindley PJ, Bethony JM, King CH, Pearce EJ, et al (2008) Helminth infections: the great neglected tropical diseases. J Clin Invest 118: 1311–1321 Available: http://www.pubmedcentral.nih.gov/articlerender.fcgi?artid=2276811&tool=pmcentrez&rendertype=abstract. Accessed 3 September 2013. 18382743 10.1172/JCI34261 PMC2276811

[2] Ingram J, Knudsen G, Lim KC, Hansell E, Sakanari J, et al (2011) Proteomic analysis of human skin treated with larval schistosome peptidases reveals distinct invasion strategies among species of blood flukes. PLoS Negl Trop Dis 5: e1337 Available: http://dx.plos.org/10.1371/journal.pntd.0001337. Accessed 8 August 2013. 21980548 10.1371/journal.pntd.0001337 PMC3181243

[3] Dresden MH, Asch HL (1972) Proteolytic enzymes in extracts of Schistosoma mansoni Cercariae. Biochim Biophys Acta 289: 378–384 Available: http://www.sciencedirect.com/science/article/pii/0005274472900897. Accessed 18 December 2013. 4346516 10.1016/0005-2744(72)90089-7

[4] Curwen RS, Ashton PD, Sundaralingam S, Wilson RA (2006) Identification of novel proteases and immunomodulators in the secretions of schistosome cercariae that facilitate host entry. Mol Cell Proteomics 5: 835–844 Available: http://www.mcponline.org/content/5/5/835.short. Accessed 18 December 2013. 16469760 10.1074/mcp.M500313-MCP200

[5] Ray D, Nelson TA, Fu C-L, Patel S, Gong DN, et al (2012) Transcriptional profiling of the bladder in urogenital schistosomiasis reveals pathways of inflammatory fibrosis and urothelial compromise. PLoS Negl Trop Dis 6: e1912 Available: http://dx.plos.org/10.1371/journal.pntd.0001912. Accessed 2 August 2013. 23209855 10.1371/journal.pntd.0001912 PMC3510078

[6] Drake L, Korchev Y, Bashford L, Djamgoz M, Wakelin D, et al (1994) The major secreted product of the whipworm, Trichuris, is a pore-forming protein. Proc Biol Sci 257: 255–261 Available: http://rspb.royalsocietypublishing.org/content/257/1350/255. Accessed 14 August 2013. 7991635 10.1098/rspb.1994.0123

[7] Drake LJ, Barker GC, Korchev Y, Lab M, Brooks H, et al (1998) Molecular and functional characterization of a recombinant protein of Trichuris trichiura. Proc Biol Sci 265: 1559–1565 Available: http://rspb.royalsocietypublishing.org/content/265/1405/1559.short. Accessed 18 December 2013. 9744108 10.1098/rspb.1998.0472 PMC1689327

[8] Barker GC, Bundy DAP (1999) Isolation of a gene family that encodes the porin-like proteins from the human parasitic nematode Trichuris trichiura. Gene 229: 131–136 Available: http://www.sciencedirect.com/science/article/pii/S0378111999000396. Accessed 18 December 2013. 10095112 10.1016/s0378-1119(99)00039-6

[9] Werneck-Silva AL, Alvares EP, Gama P, Damião AOMC, Osaki LH, et al (2006) Intestinal damage in strongyloidiasis: the imbalance between cell death and proliferation. Dig Dis Sci 51: 1063–1069 Available: http://www.ncbi.nlm.nih.gov/pubmed/16865572. Accessed 29 August 2013. 16865572 10.1007/s10620-006-8010-2

[10] Xia C-M, Zhao Y, Jiang L, Jiang J, Zhang S-C (2011) Schistosoma japonicum ova maintains epithelial barrier function during experimental colitis. World J Gastroenterol 17: 4810–4816 Available: http://www.pubmedcentral.nih.gov/articlerender.fcgi?artid=3229631&tool=pmcentrez&rendertype=abstract. Accessed 5 August 2013. 22147983 10.3748/wjg.v17.i43.4810 PMC3229631

[11] Višnjar T, Kocbek P, Kreft ME (2012) Hyperplasia as a mechanism for rapid resealing urothelial injuries and maintaining high transepithelial resistance. Histochem Cell Biol 137: 177–186 Available: http://www.ncbi.nlm.nih.gov/pubmed/22127649. Accessed 3 September 2013. 22127649 10.1007/s00418-011-0893-0

[12] Kamal M, Dehlawis MS, Rosa Brunet L, Wakelin D (2003) Paneth and intermediate cell hyperplasia induced in mice by helminth infections. Parasitology 125: 275–281 Available: http://journals.cambridge.org/abstract_S0031182002002068. Accessed 3 September 2013. 10.1017/s0031182002002068 12358424

[13] Turner JD, Narang P, Coles MC, Mountford AP (2012) Blood flukes exploit Peyer's Patch lymphoid tissue to facilitate transmission from the mammalian host. PLoS Pathog 8: e1003063 Available: http://dx.plos.org/10.1371/journal.ppat.1003063. Accessed 30 May 2013. 23308064 10.1371/journal.ppat.1003063 PMC3534376

[14] Bennuru S, Nutman TB (2009) Lymphangiogenesis and lymphatic remodeling induced by filarial parasites: implications for pathogenesis. PLoS Pathog 5: e1000688 Available: http://dx.plos.org/10.1371/journal.ppat.1000688. Accessed 9 August 2013. 20011114 10.1371/journal.ppat.1000688 PMC2781552

[15] Nelson FK (1991) The immunodeficient scid mouse as a model for human lymphatic filariasis. J Exp Med 173: 659–663 Available: http://jem.rupress.org/content/173/3/659.abstract. Accessed 3 September 2013. 1997651 10.1084/jem.173.3.659 PMC2118823

[16] Morchón R, González-Miguel J, Mellado I, Velasco S, Rodríguez-Barbero A, et al (2010) Adult Dirofilaria immitis excretory/secretory antigens upregulate the production of prostaglandin E2 and downregulate monocyte transmigration in an “in vitro” model of vascular endothelial cell cultures. Vet Parasitol 170: 331–335 Available: http://dx.doi.org/10.1016/j.vetpar.2010.02.034. Accessed 30 August 2013. 20338689 10.1016/j.vetpar.2010.02.034

[17] Kang Y-J, Jo J-O, Cho M-K, Yu H-S, Ock MS, et al (2011) Trichinella spiralis infection induces angiogenic factor thymosin β4 expression. Vet Parasitol 181: 222–228 Available: http://dx.doi.org/10.1016/j.vetpar.2011.03.058. Accessed 21 August 2013. 21531510 10.1016/j.vetpar.2011.03.058

[18] Despommier D (1998) How Does Trichinella spiralis Make Itself at Home? Parasitol Today 14: 318–323 Available: http://www.sciencedirect.com/science/article/pii/S0169475898012873. Accessed 4 February 2014. 17040798 10.1016/s0169-4758(98)01287-3

[19] Maeda H, Wu J, Sawa T, Matsumura Y, Hori K (2000) Tumor vascular permeability and the EPR effect in macromolecular therapeutics: a review. J Control Release 65: 271–284 Available: http://www.sciencedirect.com/science/article/pii/S0168365999002485. Accessed 17 December 2013. 10699287 10.1016/s0168-3659(99)00248-5

[20] Doenhoff MJ, Hassounah O, Murare H, Bain J, Lucas S (1986) The schistosome egg granuloma: immunopathology in the cause of host protection or parasite survival? Trans R Soc Trop Med Hyg 80: 503–514 Available: http://www.ncbi.nlm.nih.gov/pubmed/3492792. Accessed 26 November 2013. 3492792 10.1016/0035-9203(86)90126-4

[21] Amiri P, Locksley RM, Parslow TG, Sadick M, Rector E, et al (1992) Tumour necrosis factor alpha restores granulomas and induces parasite egg-laying in schistosome-infected SCID mice. Nature 356: 604–607 Available: http://dx.doi.org/10.1038/356604a0. Accessed 18 December 2013. 1560843 10.1038/356604a0

[22] Fallon PG, Richardson EJ, Smith P, Dunne DW (2000) Elevated type 1, diminished type 2 cytokines and impaired antibody response are associated with hepatotoxicity and mortalities during Schistosoma mansoni infection of CD4-depleted mice. Eur J Immunol 30: 470–480 Available: http://onlinelibrary.wiley.com/doi/10.1002/1521-4141(200002)30:2<470::AID-IMMU470>3.0.CO;2-T/abstract. Accessed 17 December 2013. 10671202 10.1002/1521-4141(200002)30:2<470::AID-IMMU470>3.0.CO;2-T

[23] De Oliveira Fraga LA, Torrero MN, Tocheva AS, Mitre E, Davies SJ (2010) Induction of type 2 responses by schistosome worms during prepatent infection. J Infect Dis 201: 464–472 Available: http://www.pubmedcentral.nih.gov/articlerender.fcgi?artid=2842083&tool=pmcentrez&rendertype=abstract. Accessed 17 December 2013. 20043751 10.1086/649841 PMC2842083

[24] Hams E, Aviello G, Fallon PG (2013) The schistosoma granuloma: friend or foe? Front Immunol 4: 89 Available: http://www.pubmedcentral.nih.gov/articlerender.fcgi?artid=3625856&tool=pmcentrez&rendertype=abstract. Accessed 22 May 2013. 23596444 10.3389/fimmu.2013.00089 PMC3625856

[25] Anthony RM, Rutitzky LI, Urban JF, Stadecker MJ, Gause WC (2007) Protective immune mechanisms in helminth infection. Nat Rev Immunol 7: 975–987 Available: http://dx.doi.org/10.1038/nri2199. Accessed 6 August 2013. 18007680 10.1038/nri2199 PMC2258092

[26] Vella AT, Hulsebosch MD, Pearce EJ (1992) Schistosoma mansoni eggs induce antigen-responsive CD44-hi T helper 2 cells and IL-4-secreting CD44-lo cells. Potential for T helper 2 subset differentiation is evident at the precursor level. J Immunol 149: 1714–1722 Available: http://www.jimmunol.org/content/149/5/1714.abstract. Accessed 18 December 2013. 1387150

[27] Wang I-C, Fan P-C, Lu S-C, Fan C-K, Su K-E (2008) Suppression of host Th1-type granulomatous inflammation by Taenia solium metacestodes is related to down-regulation of osteopontin gene expression. Int J Parasitol 38: 239–248 Available: http://dx.doi.org/10.1016/j.ijpara.2007.07.010. Accessed 30 August 2013. 17765901 10.1016/j.ijpara.2007.07.010

[28] White AC (2000) Neurocysticercosis: updates on epidemiology, pathogenesis, diagnosis, and management. Annu Rev Med 51: 187–206 Available: http://www.annualreviews.org/doi/abs/10.1146/annurev.med.51.1.187. Accessed 6 December 2013. 10774460 10.1146/annurev.med.51.1.187

[29] García HH, Gonzalez AE, Evans CA, Gilman RH (2003) Taenia solium cysticercosis. Lancet 362: 547–556 Available: http://www.sciencedirect.com/science/article/pii/S0140673603141177. Accessed 19 December 2013. 12932389 10.1016/S0140-6736(03)14117-7 PMC3103219

[30] Rabinovich GA, Gabrilovich D, Sotomayor EM (2007) Immunosuppressive strategies that are mediated by tumor cells. Annu Rev Immunol 25: 267–296 Available: http://www.annualreviews.org/doi/full/10.1146/annurev.immunol.25.022106.141609. Accessed 12 December 2013. 17134371 10.1146/annurev.immunol.25.022106.141609 PMC2895922

